# Hemodynamic Instability from Cement Pulmonary Embolism Following Vertebroplasty: A Case Report

**DOI:** 10.3390/reports8030172

**Published:** 2025-09-07

**Authors:** Bogdan Opriță, Georgiana-Loredana Ghinea, Alexandru-Bogdan Dinu, Ruxandra Opriță

**Affiliations:** 1 Faculty of Medicine, ”Carol Davila” University of Medicine and Pharmacy, 050474 Bucharest, Romania; bogdan.oprita@umfcd.ro (B.O.); dinubogdan@gmail.com (A.-B.D.); ruxandra.oprita@umfcd.ro (R.O.); 2 Department of Emergency Medicine, Clinical Emergency Hospital of Bucharest, 014461 Bucharest, Romania; 3 Department of Gastroenterology, Clinical Emergency Hospital of Bucharest, 014461 Bucharest, Romania

**Keywords:** vertebroplasty, cement embolism, hemodynamic instability

## Abstract

**Background and Clinical Significance**: Percutaneous vertebroplasty is an effective procedure for patients with osteoporosis and fractures. However, notable side effects may occur. Cement leakage into the vascular system may be incidental, with effects ranging from asymptomatic to life-threatening conditions. The treatment of extravasation of the cement and pulmonary embolism does not have definitive guidelines and requires specific treatment for every patient, ranging from basic anticoagulation to surgical procedures. Cement embolisms without periprocedural complications—such as cardiac perforation or massive pulmonary embolism—are often stable. However, symptomatic presentations with hemodynamic instability can occur. We report a clinically significant case of symptomatic cement pulmonary embolism resulting in shock. **Case Presentation:** A 68-year-old female patient with osteoporosis and a history of cement vertebroplasty two weeks prior to admission for vertebral compression fracture arrived with a three-day history of left leg swelling and shortness of breath. Vital signs revealed hypotension and the lab tests showed elevated lactate and D-dimer, mild leucocystosis, normal PCT and a threefold increase in CRP. The ultrasound confirmed complete thrombosis of the left external iliac and common femoral vein. The thoraco-abdominal CT demonstrated the extravasation of the cement from vertebroplasty to the inferior vena cava, lumbar veins, coupled with multiple cement structures in the segmental lobar pulmonary arteries. The echocardiography showed preserved right ventricular function. The management included intravenous fluids, anticoagulation and norepinephrine. **Conclusions:** This case underlines that cement pulmonary embolism following vertebroplasty, while typically undetected, can result in significant hemodynamic compromise even in the absence of right heart failure, potentially mediated by an inflammatory response. Importantly, it highlights the possibility of delayed clinical deterioration, with instability manifesting two weeks post-procedure—distinct from the more commonly observed immediate peri-procedural complications or other stable delayed presentation.

## 1. Introduction and Clinical Significance

Percutaneous vertebroplasty is a minimally invasive procedure that is used to strengthen the bones and relieve pain by inserting cement into the vertebrae [1]. The effect of extravasation of the cement may range from asymptomatic to life-threatening. Cement pulmonary embolism occurs in 4.6% patients after vertebroplasty [2]. Some of the mechanisms that lead to cement embolism include the insufficient polymerisation of the cement, resulting in a liquid which migrates into the veins; the inadequate position of the needle and/or the excessive use of pressure; or overfilling the vertebrae, which leads to cortical destruction [3,4,5].

The management of cement extravasation and associated pulmonary embolism remains largely guided by case-reports and continues to represent a significant clinical challenge, due to the absence of standardized clinical guidelines. Consequently, this paucity of standardized protocols leads to considerable variation in clinical decision-making and therapeutic strategies. The management ranges from conservative observation in asymptomatic patients with peripheral emboli to the initiation of anticoagulation therapy (which is based typically on the clinician’s judgment and experience rather than definitive evidence), or, in selected, severe cases, surgical intervention [6]. The heterogeneity of clinical presentations, along with the absence of consensus on optimal treatment modalities, underlines the need for further research and the development of standardized clinical guidelines to support evidence-based management of this rare, but potentially serious complication.

The case below presents several distinct features not commonly described in the literature. Notably, the patient developed acute hemodynamic instability 14 days after vertebroplasty, without accompanying respiratory distress—a presentation that contrasts with previously reported cases, which typically occurred in the immediate postprocedural period and were frequently associated with respiratory failure that lead to the need of requiring mechanical ventilation [1,5]. Additionally, anticoagulation with heparin was ineffective, with variable activated partial thromboplastin time values necessitating a switch to a now oral anticoagulant, differing from other cases in which heparin demonstrated therapeutic success [1,5]. Right ventricular dysfunction and pulmonary hypertension were excluded through echocardiographic assessment, in contrast to reports of early postoperative pulmonary pressure elevation [7] and late-onset right heart strain [8]. These findings, combined with elevated inflammatory markers and the exclusion of sepsis or right ventricular strain causes of hemodynamic instability, support a potential inflammatory vascular response to bone cement as the underlying mechanism [9,10].

## 2. Case Presentation

**Figure 1:**
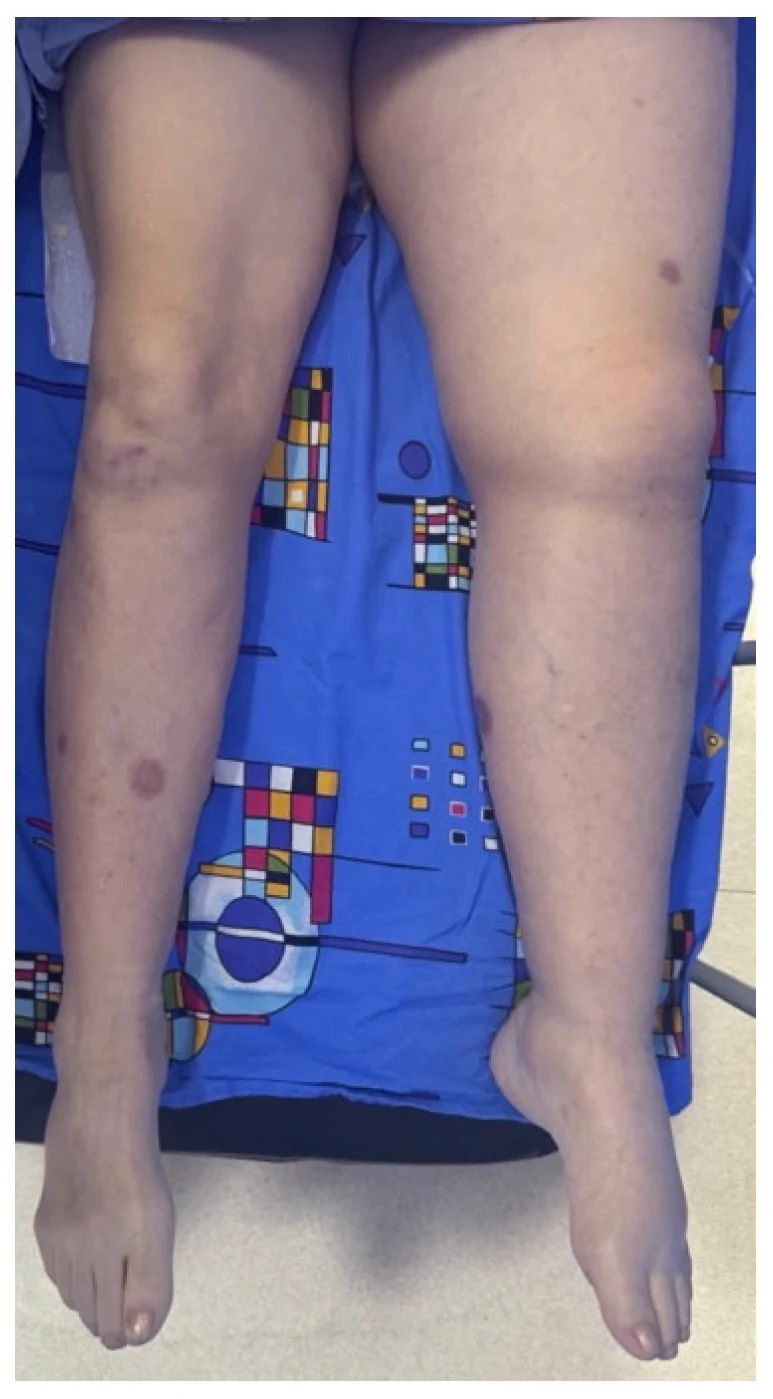
Patient presented with swelling in the patient’s left leg.

**Figure 2:**
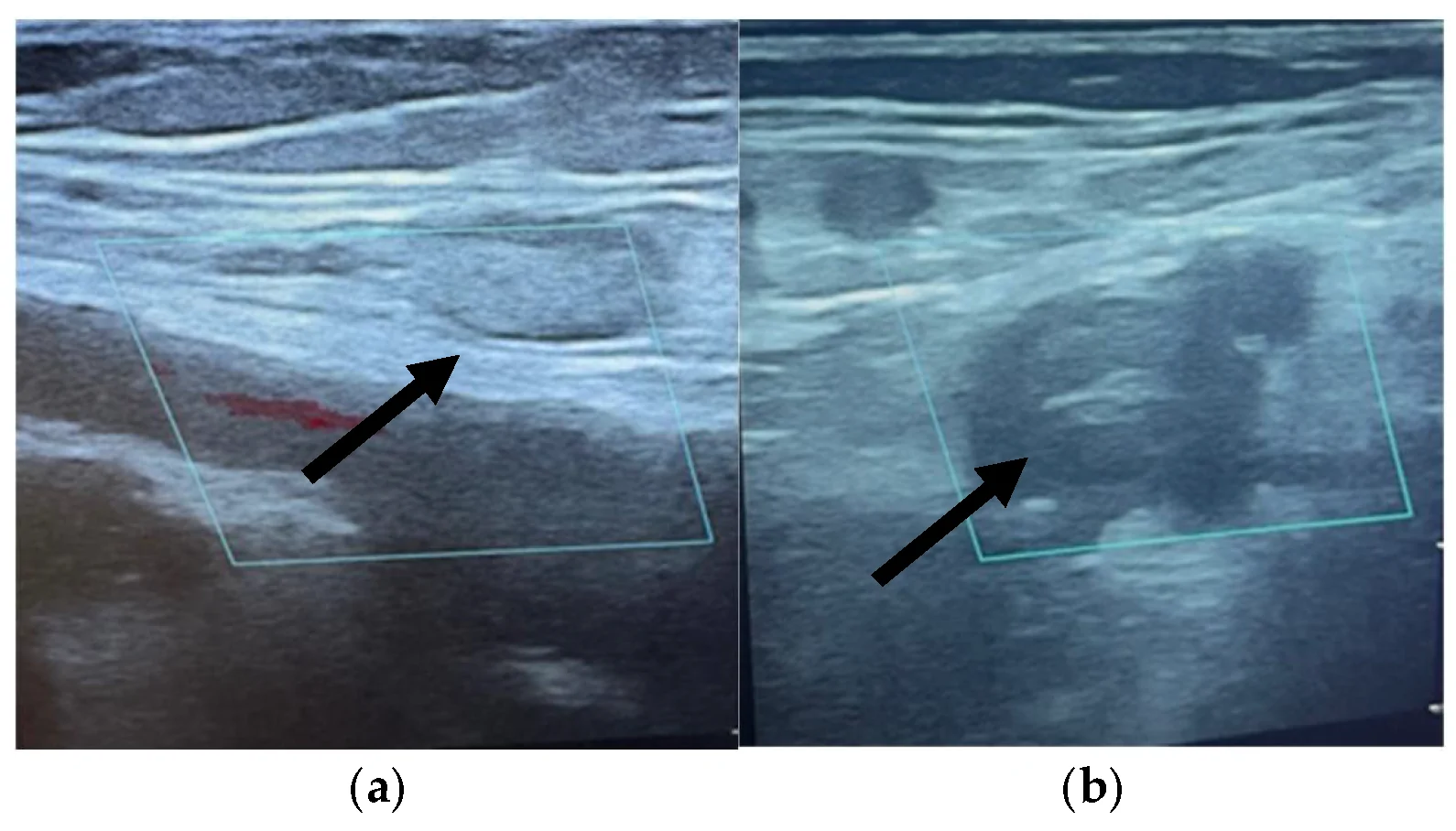
Ultrasound imaging reveals a complete (100%) thrombotic occlusion within the venous system, as demonstrated by the black arrows. (**a**) Ultrasound demonstrates a complete occlusive venous thrombus, highlighted by the arrow. (**b**) Arrow indicates the presence of an iliofemoral thrombus on ultrasound.

A 68-year-old female patient was admitted to the Emergency Department with a three-day history of swelling in the left leg (Figure 1) and shortness of breath. Her medical history includes hypertension, osteoporosis and multiple episodes of cement vertebroplasty two weeks prior to admission, for vertebral compression fracture L3, L4, and L5, as well as a history of anemia. Upon presentation, the patient was alert and conscious. Her vital signs included a blood pressure of 60/40 mmHg, heart rate of 94/min and Sp0_2_ of 93%. The electrocardiogram showed sinus rhythm and no ST-T segment abnormalities. Laboratory results revealed the following: pH 7.37, PaCO_2_ 28 mmHg, Pa0_2_ 71 mmHg, HC0_3_ 18.8 mmol/L, Lactate 3.6 mmol/L, D-dimers 760 ng/mL, CRP = 39.61 mcg/mL, PCT = 0.46 ng/mL, WBC = 11.67 × 10^3^/µL, Creatinine 1.91 mg/dL, INR 1.23, Hemoglobin = 10.7 g/dL, and potassium 3.1 mmol/L. Initially, the primary suspicion was deep vein thrombosis with unstable pulmonary embolism. A venous ultrasound Doppler confirmed 100% thrombosis of the left external iliac vein and common femoral vein (Figure 2). The transthoracic echocardiogram revealed ejection fraction of 55%, with no evidence of hypokinesia, strain or dilation of the right ventricle.

The patient received 2 L of crystalloids, but despite adequate volume resuscitation, norepinephrine administration was required. The norepinephrine was needed for a span of 3 days to maintain a mean arterial pressure above 65 mmHg.

**Figure 3:**
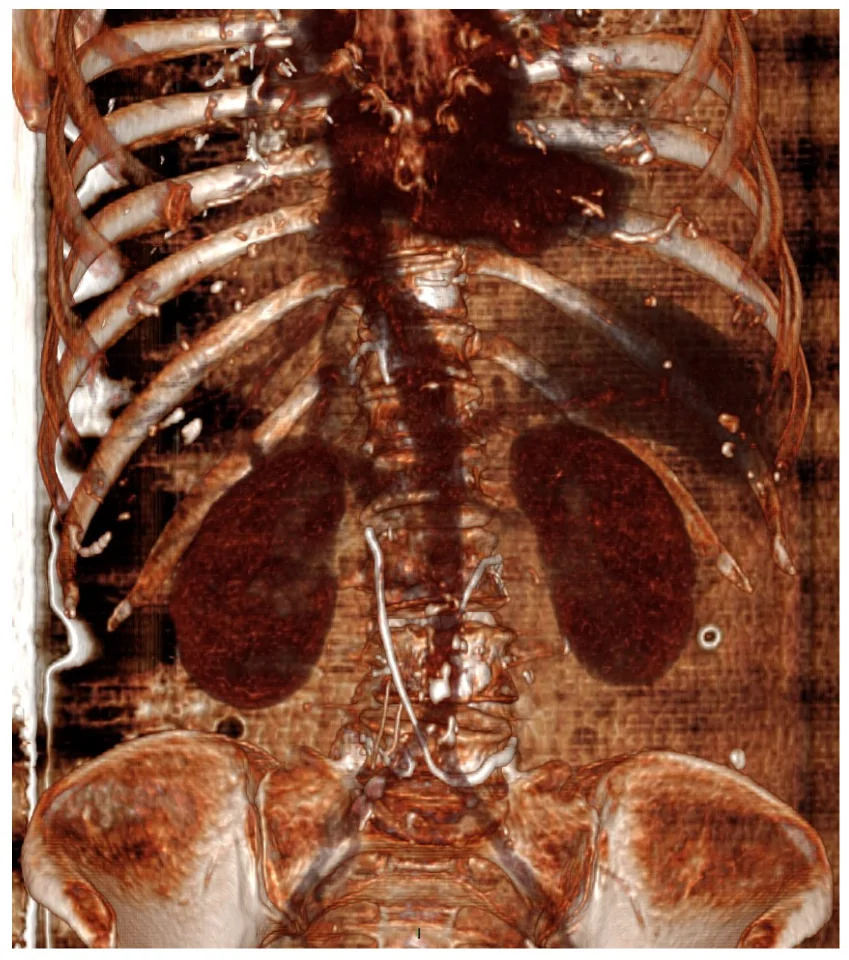
Reconstruction CT: Cement emboli originating from a lumbar vein followed an oblique rightward and cranial course, extending into a tubular structure in the inferior vena cava.

**Figure 4:**
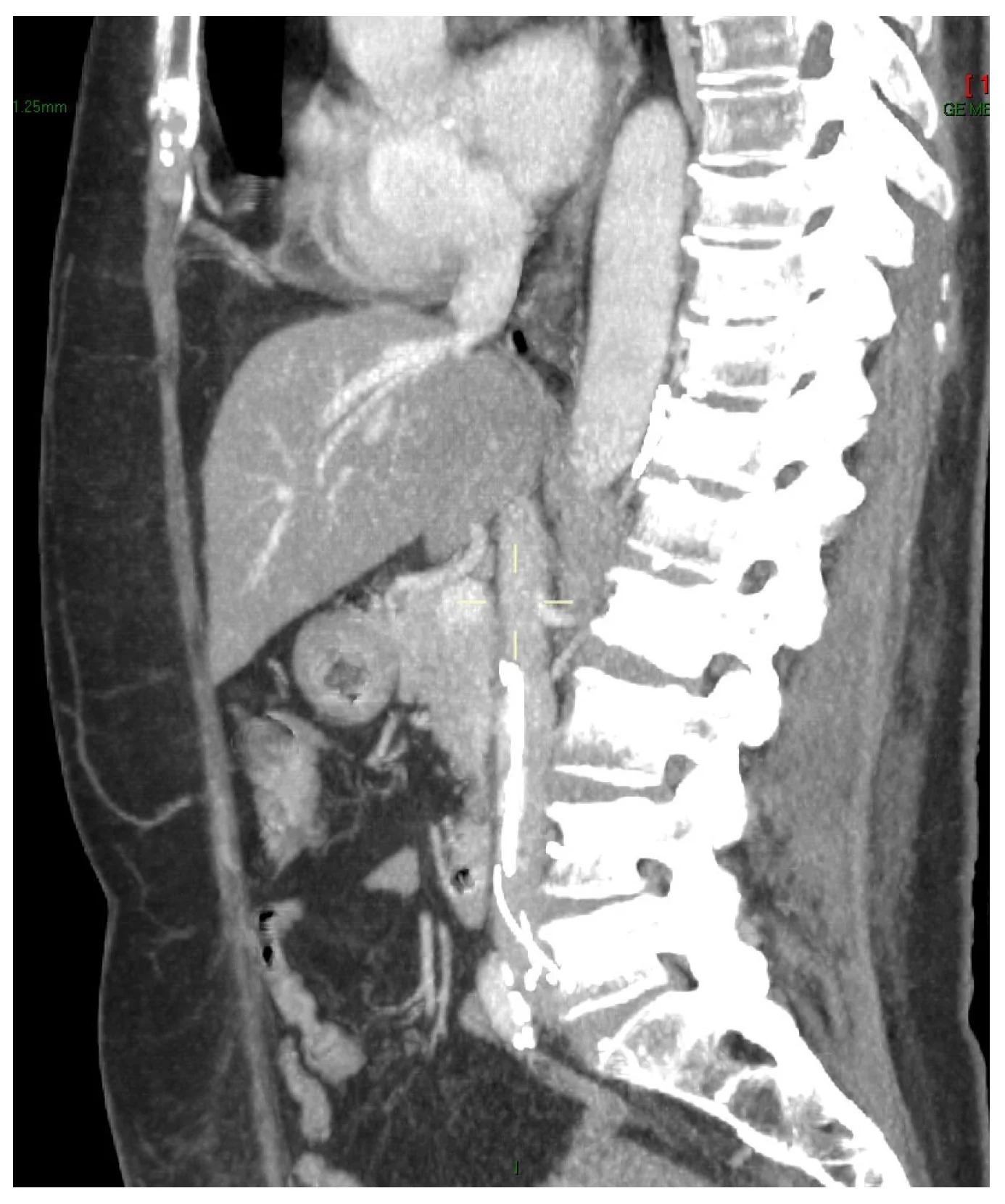
A tubular cement embolus is present within the lumen of the inferior vena cava, caudal to the point of the renal veins.

**Figure 5:**
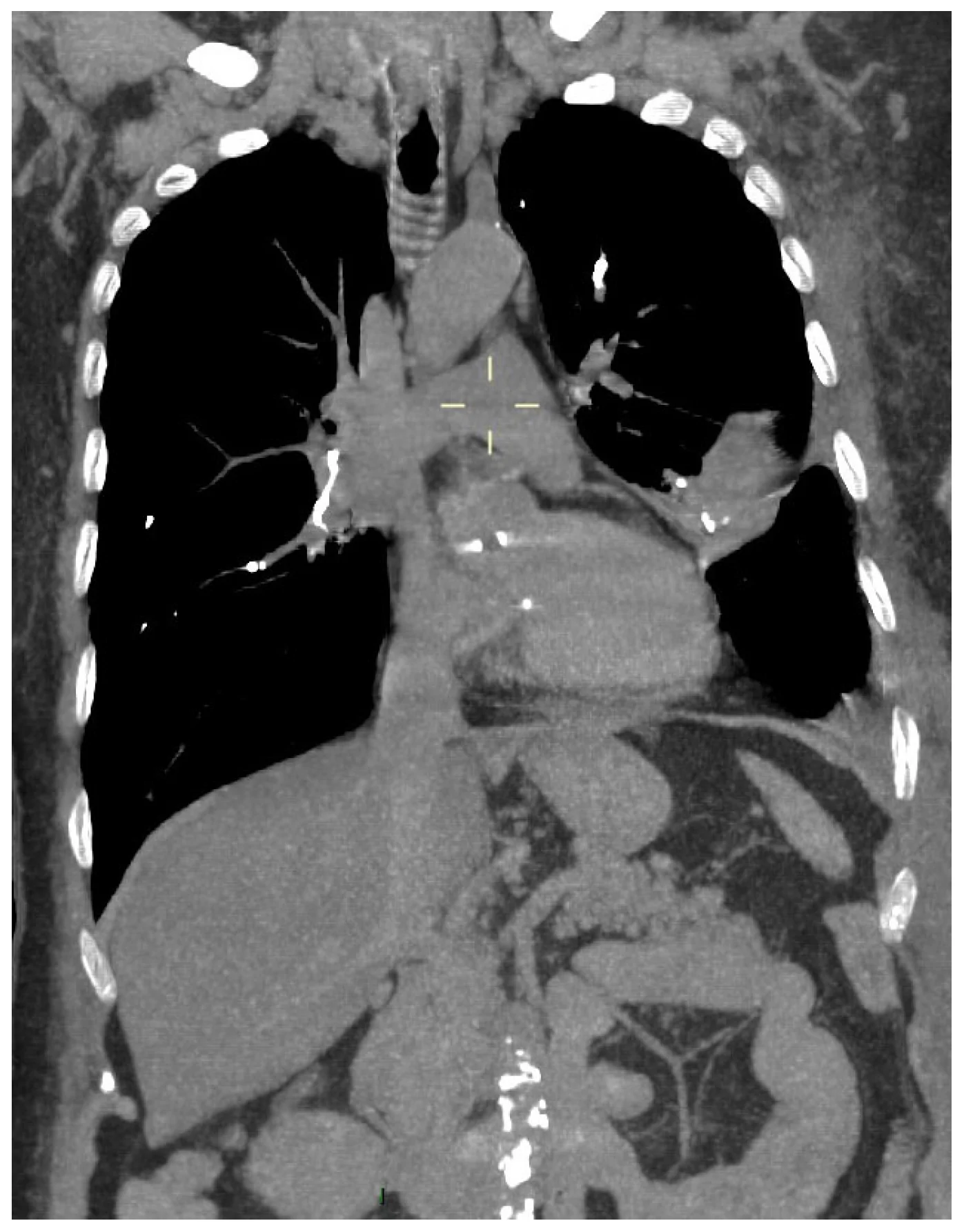
Cement emboli are present in the pulmonary arteries and lumbar vertebrae.

**Figure 6:**
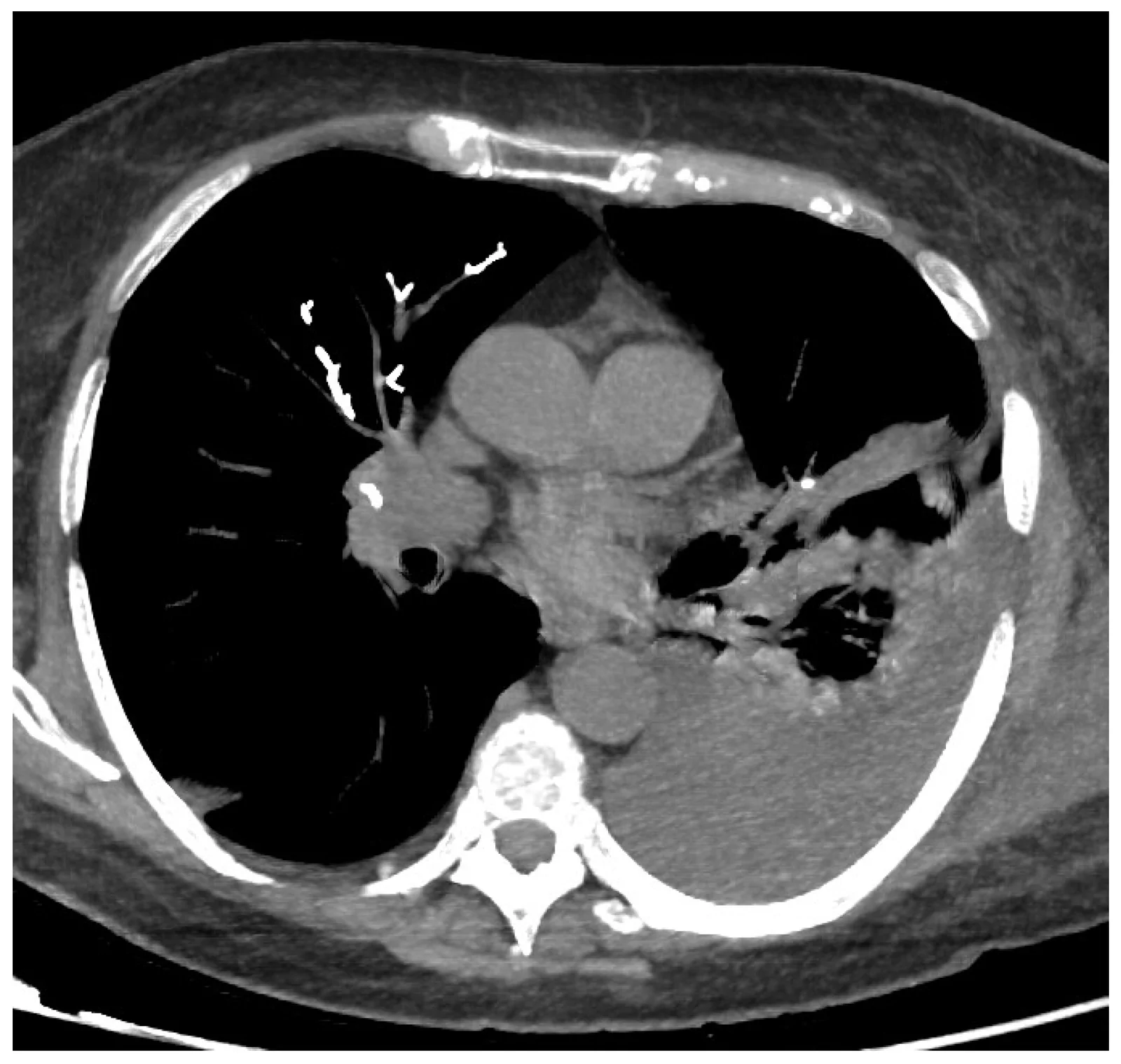
Evidence of cement emboli is noted within the pulmonary arteries.

A CT scan of the thorax, abdomen and pelvis was conducted. The scan showed tubular densities of cement in the inferior vena cava from the level of the renal veins to the lumbar vein and also multiple cement structures in the segmental lobar arteries (Figure 3, Figure 4, Figure 5 and Figure 6).

The patient started receiving anticoagulation with Heparin for an APTT of 60–80 s. Furthermore, she was consulted by a cardiovascular surgeon and radiological interventionist for placing a vena cava filter or a surgical embolectomy, but the risks were believed to outweigh the benefits and it was determined that such procedures may be considered in the future if further complications occurred.

Considering the presence of intravascular foreign material and the serologic evidence of inflammation, empirical antibiotic therapy with Piperacillin-Tazobactam was initiated for a period of nine days. Clinical progression under treatment remained stable, with persistent oedema of the left lower limb and no significant improvement, accompanied by uncontrolled APTT values fluctuating between sub-therapeutic and supra-therapeutic levels. Consequently, anticoagulation therapy was switched to a novel oral anticoagulant (Apixaban).

Follow-up CT imaging revealed the development of a large left pleural effusion, while other radiological densities remained stable. Thoracentesis with pleurostomy placement was performed, resulting in the evacuation of 1700 mL of transudate fluid. Continued pleural drainage and serial chest radiographs demonstrated the resolution of the pleural effusion, allowing for removal of the pleural drain. Bacteriological examination of the pleural fluid identified *Streptococcus viridans* in an afebrile patient with improved general condition and decreasing inflammatory markers, in the absence of antibiotic therapy and with clear pleural fluid, suggesting probable contamination of the samples. Clinical progression was favourable, with significant improvement in left lower limb oedema and resumption of active mobilization.

The patient was discharged with the recommendation of oral anticoagulation with apixaban with follow up evaluation after 6 months.

## 3. Discussion

Osteoporosis in elderly individuals represents a significant contributing factor in the development and pathogenesis of vertebral fractures. Among the available treatment options, vertebroplasty involving the injection of bone cement has shown potential to be an effective therapeutic approach for managing vertebral fractures, particularly in terms of providing relief from pain. In addition to alleviating pain, this procedure may also contribute to an improvement in functional autonomy, allowing patients to regain mobility and independence in their daily activities. However, it is important to note that one of the potential complications associated with this procedure is cement embolism, which can display a wide spectrum of clinical manifestations, ranging from asymptomatic to severe, potentially fatal outcomes [11,12,13].

Surgical embolectomy may serve as a definitive and potentially curative intervention for the removal of embolized cement material. However, this procedure is associated with several inherent risks and potential complications including pulmonary artery injury, massive bleeding or distal embolization. Despite these concerns, surgical embolectomy may represent the only viable therapeutic option in certain critical situations—particularly when cement fragments have migrated into the arterial system, as documented in prior cases [14]. In instances where the cement embolism poses an immediate threat to the patient’s life, cardiac surgery may also become necessary as an emergency life-saving measure [15].

The absence of specific guidelines for treatment is due to the lack of evidence-based studies on the topic. As such, recommendations are solely based on case reports. The treatment may involve anticoagulation therapy and although the cement itself is not a thrombus and cannot be dissolved, its presence may promote further thrombosis and induce activation of the cascade coagulation due to the foreign body effect and endothelial damage [13].

Our case presents several particularities not described in the literature. First of all, the patient presented herself to the emergency department 14 days after the vertebroplasty with an acute clinical condition of hemodynamic instability but without respiratory distress. Most of the acute presentations were periprocedural or in the first hours after vertebroplasty and exhibited severe respiratory distress that required mechanical ventilation [1]. One case study reports a patient who was admitted on the second day following the vertebroplasty with resting hypoxemia, presenting an oxygen saturation as low as 79% on room air. The patient was treated with anticoagulation and supplemental oxygen, and was discharged the following day on home oxygen therapy [5].

Another particular aspect of our reported case is that the patient did not show a favourable clinical response to heparin therapy, with fluctuating APTT values, which required switching to a NOAC-type anticoagulant. This is in contrast to other case reports in which heparin treatment proved to be effective [1,5].

Sepsis was ruled out as the etiology of the observed hemodynamic instability both clinically—by the absence of an infectious source, fever, tachycardia, or tachypnea—and biochemically—through negative aerobic and anaerobic blood cultures—and only mildly elevated leukocyte count, normal procalcitonin and platelet values, and preserved renal, hepatic, and coagulation functions were identified. The elevated lactate level was attributed to tissue hypoperfusion secondary to prolonged arterial hypotension. Also, the transudate pleural fluid developed after 12 days of hospitalization and was not responsible for the initial hemodynamic instability.

Also in our case, the echocardiogram excluded the cause of the hemodynamic instability as being acute right heart failure with no signs of pulmonary hypertension. This contrasts with some reports describing patients who developed elevated pulmonary artery systolic pressures immediately postoperatively [7]. Even in cases with delayed presentation, such as eight months after the procedure, the right heart strain was diagnosed based on biomarkers and imaging [8].

Several studies associate these physiological changes with right ventricular dysfunction caused by elevated pulmonary vascular resistance, which increases afterload and consequently reduces right ventricular filling and overall cardiac output. However, in our case, no significant right ventricular strain was observed, suggesting that the clinical presentation may instead be related to systemic absorption of volatile monomers or cement deposition within the vasculature, potentially impairing vascular autoregulation [9]. Additionally, some substances such as 6-keto PGF 1 α and tissue thromboplastin can lower the systemic vascular resistance, likely by triggering the release of other molecules [9]. Some findings indicate that introducing acrylic bone cement into the femur can lead to elevated levels of histamine in the plasma [10]. After we excluded all the other causes of the hemodynamic instability, we considered that the instability may be caused by an inflammatory response of the cement in the vessels, as demonstrated by a threefold increase in the value of CRP.

## 4. Conclusions

We present this case to highlight the importance of emergency physicians maintaining a high degree of suspicion for complications related to procedures such as vertebroplasty, which, although generally considered a safe treatment for osteoporotic vertebral fractures, may lead to serious adverse events. Given the increasing prevalence of osteoporosis due to population aging, clinicians should be mindful of the possibility and well-versed in the recognition and management of deep vein thrombosis and pulmonary cement embolism.

## Data Availability

The original contributions presented in this study are included in the article. Further inquiries can be directed to the corresponding author.

## Abbreviations

The following abbreviations are used in this manuscript:

PaCO_2_	Partial pressure of carbon dioxide in arterial blood
Pa0_2_	Partial pressure of oxygen in arterial blood
CRP	C-reactive protein
APTT	Activated partial thromboplastin time
PCT	Procalcitonin
